# Spoligotyping based genetic diversity of *Mycobacterium tuberculosis* in Ethiopia: a systematic review

**DOI:** 10.1186/s12879-018-3046-4

**Published:** 2018-03-27

**Authors:** Begna Tulu, Gobena Ameni

**Affiliations:** 1 Aklilu Lemma Institute of Pathobiology, Addis Ababa University, P. O. Box 1176, Addis Ababa, Ethiopia; 2 Department of Microbiology, Immunology and Parasitology, Bahir Dar University, P. O. Box 79, Bahir Dar, Ethiopia

**Keywords:** *Mycobacterium tuberculosis*, Molecular epidemiology, Genotyping, Diversity, Ethiopia

## Abstract

**Background:**

Understanding the types of strains and lineages of *Mycobacterium tuberculosis* (*M. tuberculosis*) circulating in a country is of paramount importance for tuberculosis (TB) control program of that country. The main aim of this study was to review and compile the results of studies conducted on strains and lineages of *M. tuberculosis* in Ethiopia.

**Methods:**

A systematic search and review of articles published on *M. tuberculosis* strains and lineages in Ethiopia were made. PubMed and Google Scholar databases were considered for the search while the keywords used were *M*. *tuberculosis*, molecular epidemiology, molecular typing spoligotyping and Ethiopia.

**Result:**

Twenty-one studies were considered in this review and a total of 3071 *M. tuberculosis* isolates and 3067 strains were included. These studies used spoligotyping and identified five lineages including Indo-Ocean, East Asian/Beijing, East African-Indian, Euro-American and Ethiopian in a proportion of 7.1%, 0.2%, 23.0%, 64.8%, and 4.1%, respectively. Thus, Euro-American was the most frequently (64.8%) occurring Lineage while East Asian was the least (0.2%) frequently occurring Lineage in the country. Surprisingly, the Ethiopian Lineage seemed to be localized to northeastern Ethiopia. In addition, the top five clades identified by this review were T, CAS, H, Manu and Ethiopian comprising of 48.0%, 23.0%, 11.0%, 6.0% and 4.1% of the strains, respectively. Furthermore, predominant shared types (spoligotype patterns) identified were SIT149, SIT53, SIT25, SIT37, and SIT21, each consisting of 420, 343, 266, 162 and 102 isolates, respectively, while, on the other hand, 15% of the strains were orphan.

**Conclusion:**

According to the summary of the results of this review, diversified strains and lineages of *M. tuberculosis* were found in Ethiopia, and the frequencies of occurrence of these strains and lineages were variable in different regions of the country. This systematic review is registered in the PRISMA with the registration number of 42017059263.

**Electronic supplementary material:**

The online version of this article (10.1186/s12879-018-3046-4) contains supplementary material, which is available to authorized users.

## Background

*M. tuberculosis*, the causative agent of TB is still the major health problem especially in low-income countries pausing significant morbidity and mortality [1, 2]. According to World Health Organization (WHO) report of 2016, globally there were an estimated 10.4 million new TB cases and 1.4 million TB deaths in the year 2015. Similarly, 11% of people who developed TB were HIV positive and 31% of TB cases in African Region was estimated to live with HIV. The report also indicated that the African Region had 26% of the world’s TB cases, but carries the most severe burden relative to population [3].

Ethiopia is also among 30 high burden countries (HBCs) and TB is one the leading causes of mortality amongst the communicable diseases in the country. In 2015, the incidence of all forms of TB was 192 per 100,000 population. Moreover, Ethiopia is also one of the high TB/HIV and multi-drug resistant TB (MDR-TB) burden countries [3]. WHO estimated that the incidence of HIV/TB co-infection was 16 per 100,000 population [3]. The emergence of drug-resistant (DR), multidrug-resistant (MDR) and extreme drug resistant (XDR) strains are the greatest threat to the TB control program [4]. In Ethiopia, based on the latest national anti-TB drug susceptibility surveillance report, a significantly higher proportion of MDR TB cases were reported among previously treated cases (17.8%) compared to newly diagnosed TB cases (2.3%) [5].

It is highlighted that, in addition to the problem associated with HIV co-infection and emergence of drug resistance, the continued TB transmission is highly linked to the missed cases or undiagnosed TB cases as a result of inefficient case detection and or poor diagnosis capacity [6], ineffective vaccine [7] and prolonged anti-TB treatment [4]. On the other hand, it is becoming clear that the outcome of TB infection is strangely diverse, ranging from active pulmonary TB to latent infection and disseminated extra-pulmonary TB [8]. This diverse TB infection outcome is attributed to the dynamics of host-pathogen-environment interaction. *M. tuberculosis* is transmitted by inhalation and once the bacterium gets access to the lung it is phagocytosed by the predominant phagocytic cells, the resident alveolar macrophages. Phagocytosis of *M. tuberculosis* by macrophages triggers the initiation and production immune response, that ultimately help in the containment of infection through granuloma formation [9, 10].

About half of the exposed individuals to *M. tuberculosis* will be infected; but only 1 in 10 of those infected develops active pulmonary diseases, suggesting that there are differences in the susceptibility or resistance to diseases development among individuals [11]. Certain clinical conditions including immunodeficiency, the presence of co-infections, malnutrition, individuals with compromised lungs, and other chronic illnesses may increase susceptibility to TB diseases compared to healthy individuals [12, 13]. Moreover, the outcome of TB infection depends on the complex interaction between the host, the agent, and the environment [14].

### Genomics of *M. tuberculosis*

Human TB is predominantly caused by *M. tuberculosis* which is a member of *M. tuberculosis* complex (MTBC) know by identical 16S rRNA sequences and 99.9% similarity at nucleotide level [15]. Briefly, the members of MTBC includes *M. tuberculosis*, *M. africanum*, *M. canneti*, *M. microti*, and *M. bovis*. The members of the complex are also known by slow-growing nature with doubling time ranging from 12 to 24 h which of course affected by pathogen character and environmental factors. Despite the genotypic similarity amongst the members of MTBC, they differ greatly in terms of their ability to cause disease, host preferences, and phenotypic characteristics [15].

Based on complete genome analysis, the *M. tuberculosis* found to have close to 4.4Mbp genome and consists of 65.5% GC. Deciphering the whole genome of *M. tuberculosis* has provided new concepts on the understanding of the properties and history of the bacilli [16]. Compared to other bacteria, *M. tuberculosis* is also known by relatively highly clonal, no horizontal transfer, and low mutation and recombination rates [16].

The tendency to transmit and cause disease varies based on its phylogeny which consists of four major lineages (L-1 Indo Oceanic, L-2 East-Asian, L-3 East-African-Indian, L-4 Euro-American and more recently L-7 Ethiopian) [9, 17, 18]. For instance, the Beijing family has got a great deal of attention compared to other families of *M. tuberculosis* because some study demonstrated its association with drug resistance [19] and some experimental models have demonstrated its hypervirulence [20]. On the other hand, other human-associated *M. tuberculosis* lineages have been given less attention partly because of the lack of standardized and phylogenetically robust classification system and associated nomenclature [18].

Molecular typing of *M. tuberculosis* is applied to study the type of strain circulating, distinguishing relapse strain versus re-infection, or detecting laboratory cross-contamination of *M. tuberculosis* strain [18] and overall evaluating the TB control programmes. With this aim, the currently available *M. tuberculosis* genotyping tools are not equally appropriate. For instance, restriction fragment length polymorphism (RFLP) analysis which bases on the monitoring the number of insertion sequence IS6110 in the chromosome which varies among different strains [21] and mycobacterial interspersed repetitive units-variable tandem repeats of DNA tandem repeats (MIRU-VNTRs) which rely on measuring repetitive DNA elements [22] are tools known by having relatively highly discriminatory power compared to spoligotyping that detects polymorphisms present in a direct repeat (DR) locus [23]. Although spoligotyping is also prone to homoplasy as individual spacers can be deleted independently in phylogenetically unrelated strains, it has several large international databases that compiled thousands of clinical isolates from many countries [18, 24].

Most recently, large sequence polymorphisms (LSPs) and single nucleotide polymorphisms (SNPs) have been introduced primarily to study the phylogenetic and strain classification of *M. tuberculosis* [24]. Above all, whole genome sequencing (WGS) is hoped to be the next gold standard for molecular epidemiology of MTBC because it has demonstrated a much higher discriminatory power than the standard genotyping tools despite its high cost and requirement of bioinformatics capacity to analyze the data [18].

In Ethiopia, there are several studies conducted to understand the transmission dynamics and types of stains involved in causing TB [17, 25–52]. However, the majority of these reports use different methodologies and as a result, it is difficult to understand the clear picture of transmission dynamics and strains involved in causing TB in the country. And also a systematic review of current genotypes has not been performed before. Hence, in order to analyze the circulating *M. tuberculosis* strains from Ethiopia, we reviewed studies that determined the genetic diversity of *M. tuberculosis* strains in patients with either pulmonary TB or extra-pulmonary TB from different Regions of Ethiopia.

## Methods

PubMed and Google Scholar Databases were used to identify studies with no limitation to the language and year of publication. The last search was conducted on March 27, 2017, using terms: *Mycobacterium tuberculosis* AND molecular epidemiology OR molecular typing OR spoligotyping AND Ethiopia. The analysis was performed based on the Preferred Reporting Items for Systematic reviews and Meta-Analysis (PRISMA) Statement [53].

The screening of articles was performed based on their relevance of the title, abstract and manuscript review. In order to minimize the risk of bias, the data was extracted from the selected studies and inserted into a data sheet by one reviewer and across studies, genotype frequency comparison was performed only when the same genotyping method was used.

This review was conducted on studies published on genetic diversity of *M. tuberculosis* isolates from different regions of the Ethiopian population. Ethiopia is a country situated between 8^0^N and 38°E coordinates in the Horn of Africa. The country shares border with Kenya to the south, Eritrea to the north and northeast, Djibouti and Somalia to the east and Sudan and South Sudan to the west. The Country is one of the most populous landlocked country in the world, as well as the second most populous country in Africa next to Nigeria with a population close to 100 million people. The country is also known by a home diverse nation and nationalities with more than 80 ethnolinguistic groups. The three largest nations are Oromo, Amhara, and Somali (https://en.wikipedia.org/wiki/Ethiopia).

The following major information was extracted from each study: 1) type of study participants (including pulmonary or extrapulmonary TB from Ethiopian regions, whether the participants were primary TB patients or re-treatment patients); 2) characteristics of genetic diversity (including region, year, number of strains and population); 3) type of intervention (type of genotyping method used: spoligotyping); 4) type of outcome measures (clustering rate, shared types, lineages, and frequency of novel genotypes).

Three different analysis was performed. First, we obtained the frequency of shared types found in each study and compared it to Ethiopian regions. Clustering rates were also determined (clustered shared type, representing two or more identical shared type, representing two or more identical shared type found within study/region; unique, representing a single shared type found within study/region). Moreover, a multi-marker database for *M. tuberculosis* (SITVITWEB) and TBinsight were also used to supplement for those studies which do not include spoligotype description and frequency.

## Result

**Fig. 1:**
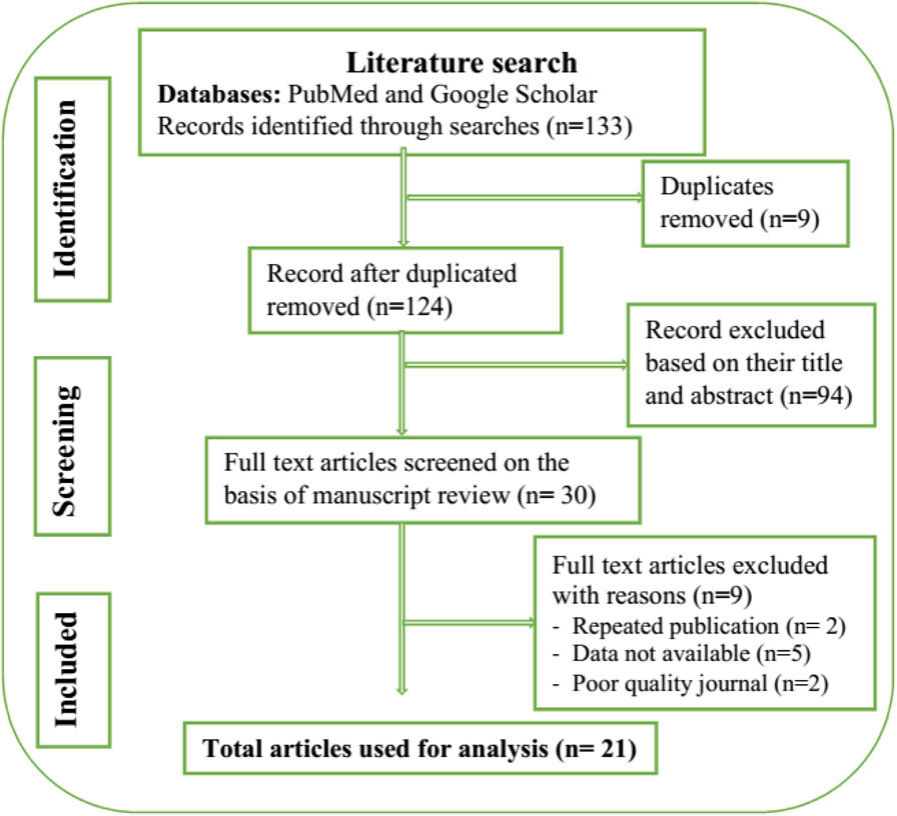
PRISMA flow diagram of study selection and literature search. A total of 21 studies involving 3067 clinical strains were identified for inclusion in the review. A total of 133 citations were identified from PubMed and Google Scholar databases. Of these, 30 studies were selected which used genotyping analysis by Spoligotyping after reviewing the title and abstracts. Additional screening was conducted in the full text and journal quality was considered and additionally, 9 studies were excluded because of repeated publication and data were not available

We performed a qualitative synthesis of the results for the genotyping and their outcome measures because the studies we found varied significantly based on the study design used, types of study participants and methods used for genotyping. As a result, the scope of this review primarily focuses on the results of the studies, their applicability and their limitations rather than meta-analysis. The main inclusion criteria used were entitled strains from Ethiopia with genetic diversity analysis. We selected studies published in English from Ethiopia without any time limitations. A total of 21 studies were selected for the purpose of this review (Fig. 1). In these studies, a total of 11,365 TB patients were involved and 3067 *M. tuberculosis* strains were obtained.

In understanding strain diversity of *M. tuberculosis*, whole genome sequencing is believed to offer many advantages compared to the classical methods like RFLP, spoligotyping and MIRU-VNTR studies [18]. Nevertheless, whole genome sequencing remains relatively costly and requires advanced capacity ranging from wet lab to big data analysis [18]. In line with this, there were only a few studies reported genome sequencing of *M. tuberculosis* and even MIRU-VNTR techniques in Ethiopia [17, 41, 50]. Recently, Firdessa et al. sequenced four *M. tuberculosis* isolates out of 36 isolates with unusual pattern (missing spacers 4–24) using (Illumina Inc., San Diego, CA, USA) and found to be members of L-7 localized between ancient lineage 1 and modern lineages 2, 3 and 4 of *M. tuberculosis* phylogeny [17]. Similarly, 30 L-7 strains from the Northern part of Ethiopia were sequenced and identified over 800 mutations specific to the lineage with a total of 22,346 bp deletions [50].

Most recently, Comas et al. sequenced 66 strains [41] from the previously spoligotyped *M. tuberculosis* collections [17] and identified representatives of five of the eight L4 sub-lineages in Ethiopian data set and assigned three of the five within L3 (provisionally named as L3.ETH1, L3.ETH2 and L3.ETH) and four of eight within L1 [41]. Likewise, strains like SIT 343, SIT 910, and SIT 1729 which are found to be assigned under new Ethiopian L-7 [17, 46] were being reported as either *M. africanum* (the West African Lineage) or Indo Oceanic or unique or new or unknown strains [30, 31, 34, 37, 39, 44, 47, 51, 52, 54].

On the other hand, the highest number of strains which were not registered in the global database (new/orphan) were reported from the study at Oromia (45.8%) [36]. A high clustering rate has been reported from Ethiopia which ranges from 31.2% [49] to 85.9% [32]. Consistently, two studies on drug-resistant strains from Addis Ababa city administration also reported very high clustering rates 80.4% [28] and 85.9% [32].

### Spoligotyping results of *M. tuberculosis* strains from Ethiopia

**Fig. 2:**
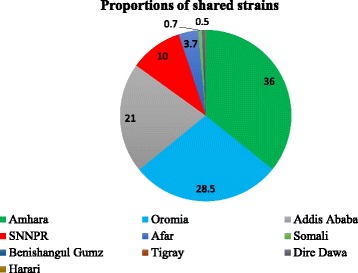
Proportions of shared strains of *M. tuberculosis* reported from Ethiopia

**Fig. 3:**
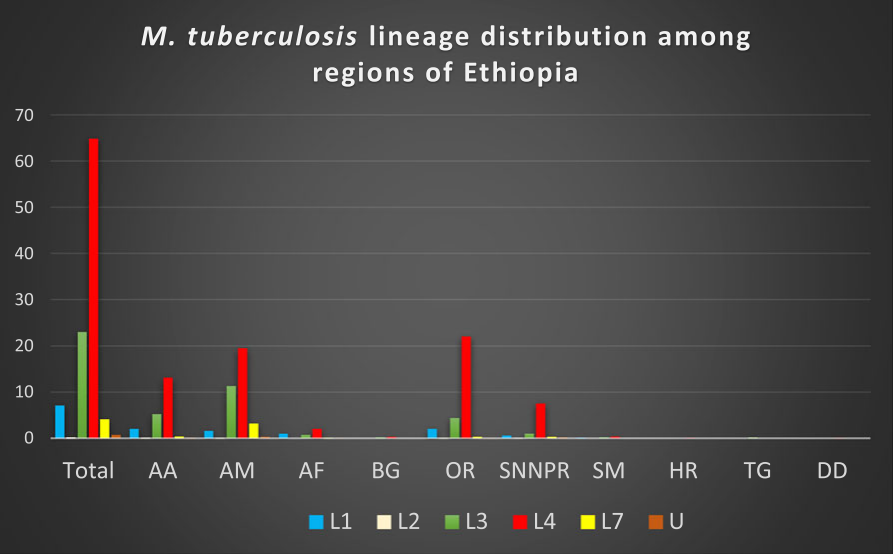
Distribution of *M. tuberculosis* lineages using spoligotyping across regions of Ethiopia. The lineages identified were: Lineage 1 (L-1) or Indo-Oceanic (IO), L-2 or East Asian (EA), L-3 or East African India (EAI) and L-4 or Euro-American (EUA), L-7 or Ethiopian and Unknown (U). The predominant lineages identified from almost all regions of the country were L-4. The Ethiopian L-7 was mainly identified from Amhara region and there was no data from Gambella region and very few strains (four) belongs to L-3 from Tigray region and one strain each belongs to L-4 from Dire Dawa city and Harari region. AA: Addis Ababa, AM: Amhara, AF: Afar, BG: Benishangul-Gumz, DD: Dire Dawa, HR: Harari, OR: Oromia, SNNPR: South Nation and Nationalities Peoples’, SM: Somali and TG: Tigray. Proportions of total lineages identified in Ethiopia namely; L-1 (7.1%), L-2 (0.2%), L-3 (23.0%), L-4 (64.8%) and L-7 (4.1%)

There were only very few studies reported using methods like RFLP-IS6110 (2 studies), WGS (3 studies) and MIRU-VNTR (3 studies). As a result, for the purpose of this review, we only included studies that reported based on the spoligotyping techniques (Table 1). A total of 21 studies have used spoligotyping for genetic diversity analysis (*n* = 3071). In the Additional file 1, a description of the *M. tuberculosis* shared types (*n* = 2596) in Ethiopia so far from nine regional states and two federal cities of Ethiopia were listed. Regarding the diversity and proportions of shared strains reported from The Regions, the highest proportion of strains were reported from Amhara region (AM) (36.0%) followed by Oromoia (OR) (28.5%), Addis Ababa (AA) city administration (21.0%), and South Nation and Nationalities Peoples Region (SNNPR) (10.0%). There were only four strains from Tigray (TG), one strain each from Dire Dawa (DD) and Harari (HR) regions reported in the shared spoligotyping pattern. Unfortunately, there were no reported shared strains from Gambella region. The largest shared spoligotyping pattern was from Amhara (AM) region followed by Oromia (OR) and Addis Ababa (AA) regions (Figs. 2 and 3). On the other hand, the total of 15.0% of the reported strains was found to be orphan.

**Table 1:** Description of reports on the strains of *M. tuberculosis* isolated from Ethiopia included in this review

Author	Study Region^a^	Type of Patients^b^	No. isolates	Typing method^c^	No. strains	Clustering rate (%)	Shared rate^d^ (%)	Predominant strains	Lineage/Clade
Agonafir et al., 2010 [28]	AA	MDR-TB	107	Spoligotyping	81	80.43	82.61	SIT149	T, CAS
Deribew et al., 2012 [29]	OR	PTB	17	Spoligotyping	17	64.71	64.71	SIT777	T, H, CAS
Mihret et al., 2012 [30]	AA	PTB	192	Spoligotyping	192	82.81	88.02	SIT149	T, CAS, H
Ameni et al., 2013 [31]	OR	PTB	139	Spoligotyping	130	71.54	84.62	SIT149, SIT53	EA, EAI
Diriba et al., 2013 [32]	AA	MDR-TB	184	Spoligotyping	184	85.87	77.72	SIT21, SIT149	EA and IO
Firdessa et al., 2013 [17]	AA, AM, OR, SNNPR	PTB, ETB	950	Spoligotyping, MIRU-VNTR, SNP	950	62.21	81.79	SIT149, SIT25	EA, EAI
Garedew et al., 2013a [33]	AM	PTB	71	Spoligotyping	96	85.42	94.79	SIT149, SIT53	EA
Garedew et al., 2013b [34]	AM	ETB	44	Spoligotyping	44	79.54	95.45	SIT54, SIT53	EA, IO
Workalemahu et al., 2013 [36]	OR	Child TB	15	Spoligotyping	24	54.17	54.17	SIT37	EA
Yimer et al., 2013 [37]	AM	PTB	237	Spoligotyping and MIRU-VNTR	237	83.54	82.70	SIT25, SIT910	CAS_DELHI
Belay et al., 2014 [38]	AM	PTB	105	Spoligotyping	103	77.67	93.20	SIT149, SIT37	T3-ETH, T3
Debebe et al., 2014 [39]	AM	PTB	118	Spoligotyping	118	83.89	89.83	SIT25 and SIT53	EA, CAS
Disassa et al., 2015 [42]	BG	PTB	33	Spoligotyping	33	45.45	75.76	SIT53	EA
Gebrezgabiher et al., 2015 [43]	SNNPR	PTB, ETB	31	Spoligotyping	31	38.71	61.29	SIT53	EA
Getahun et al., 2015 [44]	ETH	PTB	92	Spoligotyping	91	70.33	58.24	SIT53, SIT149	EA, EAI
Korma et al., 2015 [45]	AA	ETB	58	Spoligotyping	58	60.34	82.76	SIT53, SIT149	T
Maru et al., 2015 [46]	AM	PTB	118	Spoligotyping	118	73.73	90.68	SIT25, SIT53	H, T, CAS-DELHI
Nuru et al., 2015 [47]	AM	PTB, ETB	168	Spoligotyping	168	58.33	72.62	SIT289, SIT134	EA, EAI
Zewudie et al., 2016 [51]	AA	PTB, ETB	73	Spoligotyping	73	61.64	79.45	SIT149, SIT53	T, CASI-DELHI
Nuru et al., 2017 [52]	AM	PTB, ETB	38	Spoligotyping	38	36.84	68.42	SIT53, SIT289	EA, EAI
Bedawi et al., 2017 [54]	OR & SNNPR	PTB	281	Spoligotyping	281	79.36	79.72	SIT53, SIT149	EA

^a^*AA* Addis Ababa, *AM* Amhara, *AF* Afar, *BG* BenishangulGumz, *DD* Dire Dawa, *GM* Gambella, *HR* Harari, *OR* Oromia, *SNNPR* South Nation and Nationalities Peoples Region, *SM* Ethiopian Somali, *TG* Tigray. ^b^*PTB* Pulmonary Tuberculosis, *ETB* Extra-pulmonary TB. ^c^*RFLP* Restriction fragment length polymorphism, *MIRU-VNTRs* Mycobacterial interspersed repetitive units-variable tandem repeats of DNA tandem repeats. ^d^ The spoligo international type (SIT) numbers designate spoligotypes shared by two or more patient isolates

According to the spoligotyping analysis, the identified shared types of *M. tuberculosis* were classified into four major lineages namely L-1 or Indo-Oceanic, L-2 or East Asian, L-3 or East African India, L-4 or Euro-America and L-7 or Ethiopian (Fig. 3 and Table 2).

**Table 2:** Distribution of lineages/sublineages of the shared cluster containing 3 or more isolates from Ethiopia (*n* = 2584) based on spoligotyping data

Lineage	Clade/Sublineage^a^	Total strains	Percentage
Lineage 1 (Indo-Oceanic)	EAI	22	0.9
	EAI1-SOM	5	0.2
	EAI5 or EAI3	4	0.15
	EAI6-BGD1	4	0.15
	F33	9	0.35
	MANU	156	6.0
	Manu_ancestor	41	1.6
	MANU2	115	4.5
Lineage 2 (East Asian)	Beijing	5	0.2
Lineage 3 (East African-Indian)	CAS	598	23.1
	CAS	21	0.8
	CAS_DELHI	472	18.3
	CAS1_KILI	105	4.0
Lineage 4 (Euro-American)	Haarlem	281	10.1
	H1	24	0.9
	H3	188	7.3
	H4	58	2.2
	H4/Ural-1	11	0.4
	LAM	101	3.9
	LAM1	4	0.15
	LAM10	4	0.15
	LAM3 and S/Convergent	24	0.9
	LAM5	6	0.2
	LAM7_TUR	44	1.7
	LAM9	19	0.7
	S	3	0.12
	T	1247	48.3
	T	10	0.4
	T1	502	19.4
	T1 (Tuscany variant)	3	0.12
	T1/T3	5	0.2
	T2	85	3.3
	T2-T3	5	0.2
	T3	191	7.4
	T3-ETH	434	16.8
	T4	12	0.5
	X	45	1.8
	X1	40	1.6
	X2	5	0.2
Lineage 7 (Ethiopian)	Ethiopian	107	4.1
Unknown	Unknown	19	0.7
	U	8	0.31
	U (likely H)	8	0.31
	U (likely T3)	3	0.12

^a^Clade designations according to STVIT2 and TBinsight database: Beijing clade, East African-Indian (EAI) clade and its sub-lineages, Haarlem (H) clade and its sub-lineages, Latin American-Mediterranean (LAM) clade and its sub-lineages, the ancestral “Manu” family and 3 sub-lineages, the IS6110-low-binding X clade and its sub-lineage, and an ill-defined T clade with its sub-lineages, U: Unknown patterns

The majority (64.8%) of the isolates belongs to the L-4 followed by L-3 (23.0%), L-1 (7.1%) and L-7 (4.1%). The prevalence of L-2 was the lowest (0.2%) as compared to the other lineages reported (Fig. 3). Concerning the newly investigated Ethiopian lineage, although it was reported from Addis Ababa city administration, Oromia region and SNNPR, it was predominantly reported Amhara region (77%).

**Fig. 4:**
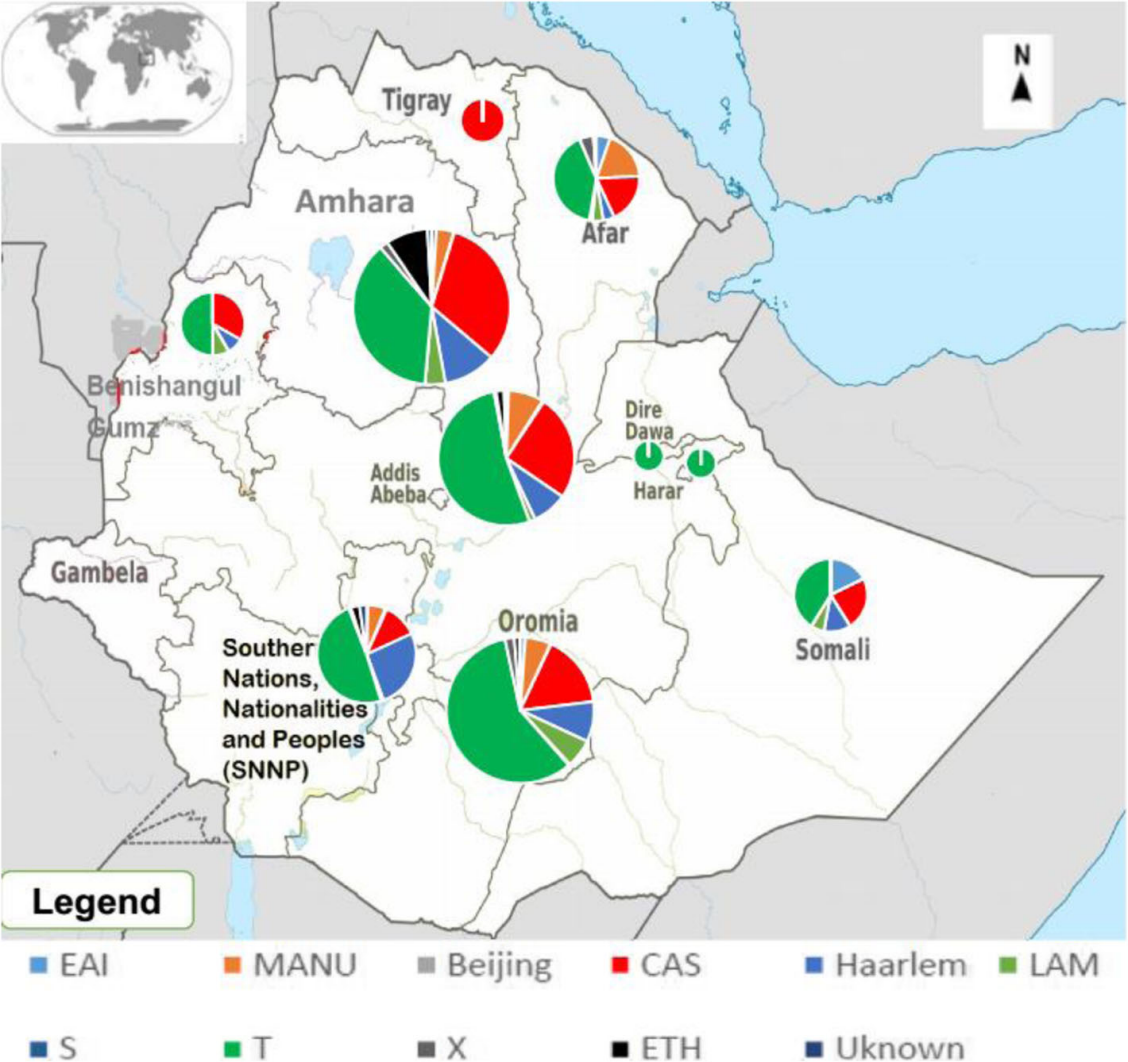
Distribution of clades of *M. tuberculosis* identified in different regions of Ethiopia. Clade designations according to STVIT2 and TBinsight database: Beijing clade in East Asian lineage; CAS clade in East African-Indian (EAI) lineage; Haarlem (H) clade, Latin American-Mediterranean (LAM) clade, the ancestral “Manu” family, an ill-defined T clade and the IS6110-low-binding X clade in Euro American; ETH belongs to the newly identified Ethiopian lineage 7

The most predominant clades identified to date include the T clade, the Central-Asian (CAS) clade, Haarlem (H) clade, Manu clade, recently investigated Ethiopian clade (Lineage 7) and Latin American-Mediterranean (LAM) clade which makes up about 96% of the reported clades so far. The T and CAS genotype shared the major clades in almost all part of the country except no information from Gambella region (Fig. 4).

The predominant associated sub-lineages identified include T1, T3-ETH, CAS-Delhi, H3, Manu2, and LAM-TUR. More specifically, the most predominant shared types were SIT149 (T3-ETH, *n* = 420, 16.2%), SIT53 (T1, *n* = 343, 13.2%), SIT25 (CAS1-Delhi, *n* = 266, 10.2%), SIT37 (T3, *n* = 162, 6.2%), SIT21 (CAS-Kili, *n* = 102, 4.0%), SIT26 (CAS-Delhi, *n* = 76, 3.0%), SIT54 (MANU2, *n* = 87, 3.4%), SIT52 (T2, *n* = 69, 2.7%), SIT910 (New Ethiopian, *n* = 65, 2.5%) and SIT289 (CAS-Delhi, *n* = 59, 2.3%) (Table 2 and Additional file 1).

In Table 3 above, a comparison of dominant isolates (> 2.0%) between our study and those previously reported in the SITVIT2 database from Ethiopia, neighboring countries in the regions and sub-regions was made. The result showed that the SIT53 (T1) and SIT26 (CAS-Delhi) were the most widely distributed isolates the regions. On the other hand, SIT910 (ETH) and 289 (CAS1-Delhi) have limited distribution in the African region (Table 3).

**Table 3:** Description of clusters containing greater than 2% of isolates in this study and their worldwide distribution in the SITVIT2 database

SIT (Clade) Octal number	N. strains (%) in this study	N. in the data base	Distribution in regions with ≥3% of a given SITs^a^	Distribution in countries with ≥3% of a given SITs^b^
149 (T3_ETH) 777,000,377,760,771	420 (16.2)	241	EURO-N 33.2, AFRI-E 32.4, EURO-W 12.4, ASIA-W 10.8, AMER-N 8.7	ETH 32.4, DNK 23.6, SAU 10.8, USA 8.7, NLD 7.1, GBR 5.8, FRA 3.7
53 (T1) 777,777,777,760,771	343 (13.2)	3812	AMER-N 21.7, EURO-W 16.1, AMER-S 9.8, EURO-S 9.2, ASIA-W 8.3, AFRI-S 6.3, EURO-N 5.5, AFRI-E 3.9	USA 21.3, ZAF 6.1, ITA 5.9, AUT 5.6, BRA 5.1, TUR 4.3, FRA 3.9, SAU 3.4
25 (CAS_DELHI) 703,777,740,003,171	266 (10.2)	254	ASIA-W 34.3, ASIA-S 11.8, EURO-N 6.3, AFRI-N 9.8, EURO-W 9.1, AMER-N 19.3	SAU 33.8, USA 19.3, IND 6.3, SDN 5.5, NLD 5.5, GBR 3.9, LIB 3.5
37 (T3) 777,737,777,760,771	162 (6.2)	243	EURO-W 18.9, AMER-N 17.3, ASIA-W 13.6, EURO-N 12.8,EURO-E 7, ASIA-S 6.2, EURO-S 4.5, AMER-S 4.1,AFRI-E 3.7, CARI 3.7	USA 15.2, SAU 9.8, DNK 7.0, NLD 5.7, AUT 4.9, CZE 4.9, IND 4.5, BGR 3.9, ITA 3.7, FRA 3.3, TUR 3.3
21 (CAS1_KILI) 703,377,400,001,771	102 (4.0)	243	AFRI-E 61.3, AMER-N 9.1, EURO-W 8.6, EURO-N 7, AFRI-S 5.3, ASIA-W 4.9	TZA 25.5, MDG 15.2, USA 9.0, ZMB 9.0, KEN 7.0, NLD 5.3, ZAF 5.3, SAU 4.9, GBR 4.1
54(MANU2) 777,777,777,763,771	87 (3.4)	114	ASIA-S 21.1, AMER-N 17.5, AFRI-S 14, ASIA-W 12.3, ASIA-N 8.8, AMER-S 3.5, EURO-N 3.5, EURO-W 3.5	IND 19.3, USA 17.5, ZAF 14.0, SAU 9.6, RUS 8.8
26(CAS1_DELHI) 703,777,740,003,771	76 (3.0)	896	ASIA-S 48.4, AMER-N 23.7, EURO-N 8.6, ASIA-W 7.9, EURO-N 3	USA 23.7, IND 23.4, PAK 16.1, BGD 8.0, SAU 7.8, NLD 4.8
52 (T2) 777,777,777,760,731	69 (2.7)	526	EURO-W 26, AMER-N 19.8, EURO-E 7.6, EURO-N 6.5, AFRI-M 6.3, ASIA-W 5.7, EURO-S 5.5, AFRI-S 3.8, AFRI-S 3.8, AFRI-E 3.2	USA 19.0, BLG 8.4, FRA 7.6, NLD 5.1, AUS 4.4, CZE 4.7, ITA 4.7, CAM 4.5, ZAF 3.8
910 (ETH) 700,000,007,177,771	65 (2.5)	5	AMER-N 60, AFRI-E 40	USA 60.0, ETH 40.0
289(CAS1_DELHI) 703,777,740,003,571	59 (2.3)	9	ASIA-S 44.4, EURO-W 22.2, ASIA-W 11.1, AUST 11.1, AMER-N 11.1	NZD 22.2, PAK 22.2, AUS 11.1, BGD 11.1, IND 11.1, SAU 11.1, USA 11.1

^a^Worldwide distribution of SITs with more than 3% in the SITVIT2 until April 2, 2017, were included. Regions and sub-regions are defined according to United Nations (http://unstats.un.org/unsd/methods/m49/m49regin.htm); regions: AFRI (Africa), AMER(Americas), ASIA(Asia), EURO(Europe), and OCE (Oceania), subdivided in: E (Eastern),M(Middle), C (Central), N (Northern), S (Southern), SE (South-Eastern), and W(Western). Furthermore, CARIB (Caribbean) belongs to Americas, while Oceania is subdivided in 4subregions, AUST (Australasia), MEL (Melanesia), MIC (Micronesia), and POLY (Polynesia).^b^The 3-letter country codes are according to http://en.wikipedia.org/wiki/ISO 3166–1 alpha-3; countrywide distribution is only shown for SITs with ≥3% of given SITs as compared to their totalnumberintheSITVIT2 database

## Discussion

*M. tuberculosis* genotypes reported from Ethiopia to date primarily belonged to the four major lineages namely; L-1 associated with populations living around the Indian Ocean, L-3 associated with populations from Central Asia also common in East Africa, L-4 associated with a widespread Euro-American lineage [41] and more recently a new L-7 belonged to Ethiopian people has been identified [17]. The investigation of the new L-7 has strongly supported the existence of TB in the area or in the continent long before the European colonial contact. Moreover, the existence of TB in the area also contributed to the rejection of the “virgin soil” hypothesis of TB in Sub-Saharan Africa. According to “virgin soil” hypothesis TB epidemic in the African region was due to European contact during the colonial period as it was originally free of TB [41].

In this review, only very few studies were found on RFLP-IS6110 [25, 26], MIRU-VNTR [35, 40, 48, 49] and genome sequencing [17, 41, 50]. As a result, the evidence generated is acceptable enough for spoligotyping. Moreover, from the reported strains so far in the country, very few strains (< 5%) were from Benishangul-Gumz region, Harari region, Tigray region and from pastoral communities of Ethiopian Somali, Oromia, and SNNPR regions. This might also compromise the complete understanding of the strain diversity and transmission dynamics of *M. tuberculosis* in Ethiopia. These regions are also known by active trans-country movements and source of refugees that could play its role in the dynamicity of TB transmission in the country. Thus, this highlights a further systematic investigation of the epidemiology of *M. tuberculosis* in the regions. Nevertheless, from the spoligotyping result, it is possible to understand that Ethiopia is a home for a complex genetic diversity of TB ranging from ancient to new or modern TB lineages.

The presence of high clustering rates for spoligotypes suggests that a high transmission rate of *M. tuberculosis* clone exists in the country. However, a study supported by Geographic Information System (GIS) mapping of cluster position of a strain reported that majority of the cluster strains are far apart [44]. Thus, GIS mapping supported cluster position studies are recommended in order to have a clear understanding of the ongoing *M. tuberculosis* transmission dynamics. The detection of a high number of previously unreported strains in the TB global databases requires further genotyping analysis to be conducted in Ethiopia.

Even though its prevalence is very low, the STI1 genotype which belongs to the Beijing genotype of East-Asian lineage has been reported from major regions of the country including Amhara, Oromia, and SNNPR and from the capital Addis Ababa. Besides its worldwide distribution, the Beijing strains have been linked to an increased virulence, drug resistance and ability to spread. Thus, it is important to monitor the distribution through continues surveillance and reporting.

### Strength and limitations

This review has explored all published articles on the genotype of M. tuberculosis identified from Ethiopian population without time and language limitation. As a result, the finding has generated a good picture of the distribution of lineages and strains across the country. However, in some regions only very few strains were reported and we found it difficult to generalize. The scope of this review also primarily focuses on the spoligotyping results of the studies and hence, its applicability is limited to spoligotyping method.

## Conclusion

This is the first systematic review of genetic diversity of *M. tuberculosis* strains from Ethiopia. The lack of genotyping information available for clinical isolates of *M. tuberculosis* from the developing regions (Benishangul-Gumz, Gambella, Afar, Ethiopian Somali) and pastoral areas of the country specifically using MIRU-VNTR genotyping and or genome sequencing requires due attention for further analysis. The detection of the high number of spoligotypes previously in the global database highlights the need for additional analysis to be conducted in Ethiopia. Most importantly, further information regarding the risk of clustering of strains using GIS supported information and the status of Beijing strains in Ethiopia needs to be monitored. Some strains are also known to be associated with drug resistance and this also requires attention in future analysis. Furthermore, additional studies are needed to evaluate the transmission dynamics and drug-resistant TB in the country.

## Additional file

Additional file 1: Description of M. tuberculosis shared types reported for TB strains isolated in Ethiopia from nine regional states and two city administrations (*n* = 2596)*.* ^a^ In the SITVIT2 database, the spoligo international type (SIT) numbers designate spoligotypes shared by two or more patient isolates. In contrast, “orphan” designates patterns reported for a single isolate. ^b^ Clade designations according to STVIT2 database: Beijing clade, East African-Indian (EAI) clade and 9 sub-lineages, Haarlem (H) clade and 3 sub-lineages, Latin American-Mediterranean (LAM) clade and 12 sub-lineages, the ancestral “Manu” family and 3 sub-lineages, the S clade, the IS6110-low-binding X clade and 3 sub-lineages, and an ill-defined T clade with 5 sub-lineages, U: Unknown patterns, ** Belongs to new Ethiopian L7. ^c^ AA: Addis Ababa; AM: Amhara; AF: Afar; BG: Benishangul Gumz; DD: Dire Dawa; GM: Gambela; HR: Harari; OR: Oromia; SNNPR: South Nation and Nationalities Peoples Region; SM: Ethiopian Somali; TG: Tigray ^d^High clustering rate reported. (DOCX 58 kb)

## Abbreviations

CAS: Central Asian
CRISPR: Clustered regularly interspaced short palindromic repeats
DR: Direct repeat
GIS: Geographic information system
HBCs: High burden countries
HIV: Human immunodeficiency virus
LAM: Latin-American
MDR: Multi-drug resistant
MIRU-VNTRs: Mycobacterial interspersed repetitive units-variable tandem repeats of DNA tandem repeats
MTBC: Mycobacterium tuberculosis complex
RFLP: Restriction fragment length polymorphism
TB: Tuberculosis
WHO: World Health Organization
XDR: Extensive drug resistant
