# Supplementary material for: Spoligotyping based genetic diversity of Mycobacterium tuberculosis in Ethiopia: a systematic review
Source: BMC Infect Dis. 2018 Mar 27;18:140. doi: 10.1186/s12879-018-3046-4 (PMC5870191; doi:10.1186/s12879-018-3046-4)
Supplement: Supplementary file 1 — Description of M. tuberculosis shared types reported for TB strains isolated in Ethiopia from nine regional states and two city administrations (n = 2596). a In the SITVIT2 database, the spoligo international type (SIT) numbers designate spoligotypes shared by two or more patient isolates. In contrast, “orphan” designates patterns reported for a single isolate. b Clade designations according to STVIT2 database: Beijing clade, East African-Indian (EAI) clade and 9 sub-lineages, Haarlem (H) clade and 3 sub-lineages, Latin American-Mediterranean (LAM) clade and 12 sub-lineages, the ancestral “Manu” family and 3 sub-lineages, the S clade, the IS6110-low-binding X clade and 3 sub-lineages, and an ill-defined T clade with 5 sub-lineages, U: Unknown patterns, ** Belongs to new Ethiopian L7. c AA: Addis Ababa; AM: Amhara; AF: Afar; BG: Benishangul Gumz; DD: Dire Dawa; GM: Gambela; HR: Harari; OR: Oromia; SNNPR: South Nation and Nationalities Peoples Region; SM: Ethiopian Somali; TG: Tigray dHigh clustering rate reported. (DOCX 58 kb) [file 12879_2018_3046_MOESM1_ESM.docx]

Additional file 1: Description of M. tuberculosis shared types reported for TB strains isolated in Ethiopia from nine regional states and two city administrations (n=2,596)

| SIT^a^ | Octal number | Clade^b^ | Total strains | % | In cluster | Regions/cities of Ethiopia^c^ | *Ref* |
| --- | --- | --- | --- | --- | --- | --- | --- |
| 1 | 000000000003771 | Beijing | 5 | 0.19 | Clustered | AA(0.4), AM(0.1), OR(0.1), SNNPR(0.4) | 3, 6, 11, 17 |
| 3 | 000000007720771 | H3 | 2 | 0.08 | Clustered | AA(0.2), SNNPR(0.4) | 3, 6 |
| 4 | 000000007760771 | LAM3 and S/Convergent | 24 | 0.92 | Clustered | AA(0.2), AM(1.0), OR(1.9), SNNPR(0.4) | 4, 6, 11, 17, 21 |
| 6 | 077777777413731 | EAI1-SOM | 1 | 0.04 | Unique | OR(0.1) | 6 |
| 10 | 477777277413771 | EAI5 or EAI3 | 2 | 0.08 | Clustered | AF(1.1), SM(5.9) | 6, 10 |
| 20 | 677777607760771 | LAM1 | 3 | 0.12 | Clustered | AM(0.1), AF(2.1) | 10, 18 |
| 21 | 703377400001771 | CAS1_KILI | 102 | 3.93 | Clustered | AA(11.0), AM(2.4), OR(2.3), SNNPR(0.4), TG(25.0) | 1, 3, 4, 5, 6, 11, 12, 15, 16, 17, 18, 19, 21 |
| 24 | 703777740003031 | CAS1_DELHI | 2 | 0.08 | Clustered | AM(0.2) | 6 |
| 25 | 703777740003171 | CAS1_DELHI | 266 | 10.25 | Clustered | AM(6.0), AM(17.0), AF(4.2), OR(7.6), SNNPR(5.1), SM(5.9) | 1, 2, 3, 4, 5, 6, 8, 10, 11, 12, 16, 17, 18, 19, 20, 21 |
| 26 | 703777740003771 | CAS1_DELHI | 76 | 2.93 | Clustered | AA(4.0), AM(2.3), A(5.3), OR(2.9), SNNPR(2.4), SM(12.0) | 1, 3, 4, 5, 6, 7, 8, 10, 11, 14, 15, 16, 18, 19, 20, 21 |
| 34 | 776377777760771 | S | 3 | 0.12 | Clustered | AF(1.1), OR(0.3) | 6 |
| 35 | 777737777420771 | H4/Ural-1 | 11 | 0.42 | Clustered | AA(0.2), AM(0.5), OR(0.3), SNNPR(1.2) | 6, 14, 17, 18 |
| 36 | 777737777720771 | H3_T3 | 2 | 0.08 | Clustered | OR(0.1), SNNPR(0.4) | 2, 6 |
| 37 | 777737777760771 | T3 | 162 | 6.24 | Clustered | AA(5.5), AM(3.1), AF(11.0), BG(8.3), OR(10.8), SNNPR(6.7) | , 2, 3, 4, 6, 8, 9, 10, 11, 12, 13, 14, 15, 16, 17, 18, 19, 20, 21 |
| 40 | 777777377760771 | T4 | 10 | 0.39 | Clustered | AA(0.7), AM(0.5), OR(0.1) | 6, 11, 17, 19 |
| 41 | 777777404760771 | LAM7_TUR | 44 | 1.69 | Clustered | AA(0.7), AM(1.5), AF(1.1), BG(8.3), OR(3.5) | 1, 3, 4, 6, 8, 10, 11, 12, 13, 18, 19, 21 |
| 42 | 777777607760771 | LAM9 | 12 | 0.46 | Clustered | AA(0.4), AM(0.8), OR(0.3), SNNPR(0.4) | 3, 6, 16, 20 |
| 43 | 777777747413771 | EAI6-BGD1 | 1 | 0.04 | Unique | OR(0.1) | 6 |
| 44 | 777777757760771 | T5 | 1 | 0.04 | Unique | AA(0.2) | 3 |
| 46 | 777777770000000 | U (likely H) | 5 | 0.19 | Clustered | AM(0.4), SNNPR(0.4) | 6, 11 |
| 47 | 777777774020771 | H1 | 11 | 0.42 | Clustered | AA(0.6), AM(0.4), OR(0.4), SNNPR(0.4) | 3, 6, 8, 11, 17, 19, 21 |
| 48 | 777777777413731 | EAI1_SOM | 4 | 0.15 | Clustered | AA(0.4), OR(0.3) | 5, 6 |
| 49 | 777777777720731 | H3 | 2 | 0.08 | Clustered | AM(0.2) | 12 |
| 50 | 777777777720771 | H3 | 35 | 1.35 | Clustered | AA(2.0), AM(1.4), AF(1.1), OR(1.2), SNNPR(0.8) | 1, 3, 4, 5, 6, 10, 11, 16, 17, 18, 20, 21 |
| 51 | 777777777760700 | T | 1 | 0.04 | Unique | AM(0.1) | 18 |
| 52 | 777777777760731 | T2 | 69 | 2.66 | Clustered | AA(2.2), AM<(3.2), AF(6.3), BG(17.0), OR(2.0), SNNPR(0.8), SM(18.0) | 1, 3, 4, 6, 7, 8, 10, 11, 13, 16, 18, 20, 21 |
| 53 | 777777777760771 | T1 | 343 | 13.21 | Clustered | AA(13.0), AM(11.0), AF(9.5), BG(25.0), OR(13.6), HR(100.0), SNNPR(23.0), SM(12.0), DR(100.0) | 1, 2, 3, 4, 5, 6, 7, 8, 9, 10, 11, 12, 13, 14, 15, 16, 17, 18, 19, 20, 21 |
| 54 | 777777777763771 | MANU2 | 87 | 3.35 | Clustered | AA(3.1), AM(2.8), AF(9.5), OR(3.8), SNNPR(3.5) | 5, 7, 8, 10, 12, 14, 16, 18, 21 |
| 56 | 777737770000000 | U (likely T3) | 3 | 0.12 | Clustered | AA(0.2), AM(0.1), OR(0.1) | 6, 11, 17 |
| 59 | 777777606060771 | LAM11-ZWE | 1 | 0.04 | Unique | OR(0.1) | 6 |
| 61 | 777777743760771 | LAM10 | 3 | 0.12 | Clustered | AM(0.3) | 16 |
| 62 | 777777774020731 | H1 | 3 | 0.12 | Clustered | AM(0.1), OR(0.3) | 6 |
| 73 | 777737777760731 | T2-T3 | 5 | 0.19 | Clustered | AA(0.2), OR(0.6) | 3, 6, 21 |
| 78 | 777777777760711 | T | 1 | 0.04 | Unique | AM(0.1) | 16 |
| 93 | 777737607760771 | LAM5 | 5 | 0.19 | Clustered | AM(0.2), OR(0.4) | 4, 6, 18, 21 |
| 100 | 777777777773771 | MANU2 | 2 | 0.08 | Clustered | AA(0.2), AF(1.1) | 5, 10 |
| 102 | 777703777760771 | T | 1 | 0.04 | Unique | OR(0.1) | 21 |
| 117 | 777767777760731 | T1 | 1 | 0.04 | Unique | AA(0.2) | 1 |
| 118 | 777767777760771 | T2 | 1 | 0.04 | Unique | OR(0.1) | 4 |
| 119 | 777776777760771 | X1 | 16 | 0.62 | Clustered | AA(0.2), AM(0.8), AF(3.2), OR(0.6), SNNPR(0.4) | 4, 6, 8, 10, 15, 21 |
| 120 | 777777577760771 | T1 | 1 | 0.04 | Unique | OR(0.1) | 9 |
| 121 | 777777775720771 | H3 | 20 | 0.77 | Clustered | AA(0.9), AM(0.5), AF(3.2), OR(1.0) | 3 |
| 131 | 777717777760771 | T1 | 2 | 0.08 | Clustered | AM(0.1), OR(0.1) | 6, 20 |
| 134 | 777777777720631 | H3 | 47 | 1.81 | Clustered | AA(0.9), AM(3.2), BG(8.3), OR(1.3), SNNPR(1.2) | 1, 3, 4, 6, 7, 11, 12, 13, 14, 18, 20, 21 |
| 137 | 777776777760601 | X2 | 4 | 0.15 | Clustered | AM(0.2), OR(0.3) | 18, 21 |
| 140 | 702777740003171 | CAS_DELHI | 1 | 0.04 | Unique | AA(0.2) | 3 |
| 141 | 703767740003771 | CAS_DELHI | 1 | 0.04 | Unique | AM(0.1) | 11 |
| 142 | 703777700003771 | CAS | 5 | 0.19 | Clustered | AA(0.4), OR(0.4) | 3, 6 |
| 149 | 777000377760771 | T3_ETH | 420 | 16.18 | Clustered | AA(23.2), AM(10.0), AF(11.0), OR(23.0), SNNPR(13.0) | 1, 2, 3, 4, 5, 6, 7, 8, 9, 10, 11, 12, 15, 16, 17, 18, 19, 20, 21 |
| 151 | 777767774020771 | H1 | 1 | 0.04 | Unique | OR(0.1) | 6 |
| 153 | 757777777760731 | T1 | 2 | 0.08 | Clustered | AM(0.2) | 12 |
| 154 | 757777777760771 | T1 | 2 | 0.08 | Clustered | AM(0.2) | 6 |
| 157 | 777737777760471 | T3 | 1 | 0.04 | Unique | OR(0.1) | 21 |
| 159 | 777740017760771 | T1 (Tuscany variant) | 3 | 0.12 | Clustered | AM(0.3) | 6, 12 |
| 168 | 777777777720671 | H3 | 5 | 0.19 | Clustered | AM(0.5) | 16, 18, 20 |
| 172 | 777777777740771 | U | 2 | 0.08 | Clustered | AA(0.1), AM(0.1) | 1, 6 |
| 173 | 777767677760771 | T1 | 5 | 0.19 | Clustered | AM(0.5) | 7, 8, 11 |
| 196 | 677777777760771 | T1 | 1 | 0.04 | Unique | AM(0.1) | 6 |
| 204 | 737777777413771 | EAI5 or EAI3 | 1 | 0.04 | Unique | SM(5.9) | 6 |
| 205 | 737777777760771 | T | 1 | 0.04 | Unique | AM(0.1) | 18 |
| 217 | 777736777760771 | T1 | 1 | 0.04 | Unique | AA(0.2) | 19 |
| 241 | 777777777760411 | T1 | 1 | 0.04 | Unique | AF(1.1) | 10 |
| 244 | 777777777760601 | T1 | 3 | 0.12 | Clustered | AA(0.2), AM(0.2) | 6 |
| 247 | 703777740003471 | CAS_DELHI | 17 | 0.65 | Clustered | AA(0.6), AM(0.6), AF(2.1), OR(0.4), TG(50.0) | 3, 6, 10, 15, 19 |
| 262 | 774777777420771 | H4 | 4 | 0.15 | Clustered | AA(0.2), AM(0.1), SNNPR(0.8) | 3, 6 |
| 272 | 037777777760771 | T1 | 5 | 0.19 | Clustered | AM(0.5) | 12 |
| 281 | 777775777760771 | T | 3 | 0.12 | Clustered | OR(0.1), SNNPR(0.8) | 14, 12 |
| 289 | 703777740003571 | CAS1_DELHI | 59 | 2.27 | Clustered | AA(0.4), AM(4.6), AF(2.1), BG(33.0), OR(0.9), SNNPR(0.4), TG(25.0) | 4, 5, 8, 9, 10, 11, 12, 15, 16, 18, 19, 20, 21 |
| 302 | 777756777760771 | X1 | 2 | 0.08 | Clustered | AM(0.2) | 16 |
| 309 | 703767740003171 | CAS1-DELHI | 1 | 0.04 | Unique | AM(0.1) | 6 |
| 333 | 777727607760771 | LAM5 | 1 | 0.04 | Unique | OR(0.1) | 6 |
| 334 | 577777777760771 | T | 1 | 0.04 | Unique | AM(0.1) | 20 |
| 336 | 777776777760731 | X1 | 18 | 0.69 | Clustered | AA(0.2), AM(0.3), AF(2.1), OR(1.5), SNNPR(0.4) | 6, 8, 10, 14, 18, 19, 21 |
| 343 | 700000007175771 | Ethiopian** | 7 | 0.27 | Clustered | AM(0.8) | 6, 16, 18 |
| 345 | 777000377760731 | T3_ETH | 5 | 0.19 | Clustered | AA(0.6), AM(0.2) | 3, 7, 8 |
| 357 | 703777740000771 | CAS1-Delhi | 5 | 0.19 | Clustered | AM(0.3), OR(0.3) | 4, 7, 8, 16, 21 |
| 358 | 717777777760771 | T1 | 5 | 0.19 | Clustered | AM(0.5) | 6, 11, 16 |
| 373 | 777777767760771 | T1 | 2 | 0.08 | Clustered | AA(0.2), AM(0.1) | 3, 12 |
| 381 | 703777740003071 | CAS1-DELHI | 1 | 0.04 | Unique | OR(0.1) | 6 |
| 393 | 777757777760771 | T1 | 2 | 0.08 | Clustered | OR(0.1), SNNPR(0.4) | 6, 12 |
| 394 | 777777777620731 | H3 | 1 | 0.04 | Unique | SNNPR(0.4) | 6 |
| 442 | 777737377760771 | T1/T3 | 5 | 0.19 | Clustered | AM(0.5) | 8, 11 |
| 462 | 777777777560771 | T1 | 10 | 0.39 | Clustered | AA(0.4), AM(0.1), OR(1.0) | 4, 6, 7, 19, 21 |
| 472 | 377777777720771 | H3 | 1 | 0.04 | Unique | OR(0.4) | 14 |
| 504 | 777737737760771 | T3 | 4 | 0.15 | Clustered | OR(0.6) | 6, 21 |
| 522 | 777777777760770 | T1 | 1 | 0.04 | Unique | AM(0.1) | 8 |
| 523 | 777777777777771 | Manu_ancestor | 41 | 1.58 | Clustered | AA(4.8), AM(0.5), AF(3.2), OR(0.7), OR(0.8) | 5, 10, 12, 18, 19, 21 |
| 565 | 777737777760671 | T3 | 2 | 0.08 | Clustered | AM(0.2) | 6, 16 |
| 584 | 777775777760731 | T2 | 12 | 0.46 | Clustered | AA(1.1), AM(0.2), OR(0.6) | 1, 3, 4, 6, 11, 16, 17, 21 |
| 591 | 777777757413771 | EAI6-BGD1 | 1 | 0.04 | Unique | OR(0.1) | 6 |
| 600 | 703777400003171 | CAS | 1 | 0.04 | Unique | OR(0.1) | 6 |
| 602 | 777777770000771 | H1 | 5 | 0.19 | Clustered | AM(0.2), OR(0.3), SNNPR(0.4) | 4, 6, 20, 21 |
| 612 | 777777777760751 | T1 | 5 | 0.19 | Clustered | AA(0.9) | 3, 17 |
| 613 | 777177777760771 | T1 | 1 | 0.04 | Unique | AM(0.1) | 12 |
| 699 | 677777777720571 | H3 | 8 | 0.31 | Clustered | AA(0.4), AM(0.1), OR(0.7) | 3, 4, 6, 18, 19, 21 |
| 747 | 777777777720471 | H3 | 1 | 0.04 | Unique | AM(0.1) | 16 |
| 750 | 37677500020771 | H3 | 1 | 0.04 | Unique | AM(0.1) | 11 |
| 751 | 77777777760771 | T | 1 | 0.04 | Unique | AM(0.1) | 16 |
| 754 | 503777740003771 | CAS1_DELHI | 1 | 0.04 | Unique | AM(0.1) | 18 |
| 764 | 777757777720771 | H3 | 9 | 0.35 | Clustered | AM(0.9), OR(0.1) | 6, 11, 12, 21 |
| 777 | 777777777420771 | H4 | 26 | 1.00 | Clustered | AA(1.8), AM(0.4), OR(0.6), SNNPR(2.4), SM(12.0) | 2, 3, 4, 5, 6, 15, 16, 17, 18, 19, 21 |
| 780 | 777777777600771 | U | 1 | 0.04 | Unique | AM(0.1) | 6 |
| 788 | 577777002060771 | T1 | 1 | 0.04 | Unique | AA(0.2) | 17 |
| 804 | 477777777760771 | T1 | 1 | 0.04 | Unique | AF(1.1) | 10 |
| 817 | 777777777420731 | H4 | 28 | 1.08 | Clustered | AA(0.6), AM(0.9), OR(0.7), SNNPR(4.7) | 2, 3, 6, 9, 12, 18, 19, 21 |
| 830 | 000000007720731 | Family36 | 1 | 0.04 | Unique | AA(0.2) | 19 |
| 866 | 577777607760771 | LAM9 | 1 | 0.04 | Unique | AA(0.2) | 17 |
| 868 | 777676777760771 | X1 | 1 | 0.04 | Unique | AM(0.1) | 8 |
| 871 | 737777777720771 | H3 | 2 | 0.08 | Clustered | AM(0.2) | 16 |
| 883 | 777777754020771 | H1 | 1 | 0.04 | Unique | AA(0.2) | 17 |
| 910 | 700000007177771 | Ethiopian** | 65 | 2.50 | Clustered | AA(1.8), AM(4.9), OR(0.7), SNNPR(1.6) | 3, 4, 6, 8, 11, 12, 15, 16, 17, 18, 19, 21 |
| 912 | 677777777413371 | F36 | 1 | 0.04 | Unique | AM(0.1) | 12 |
| 913 | 777743777760771 | T1 | 2 | 0.08 | Clustered | AM(0.1), OR(0.1) | 4, 8 |
| 924 | 777600007413371 | EAI5 or EAI3 | 1 | 0.04 | Unique | OR(0.1) | 6 |
| 926 | 773777777760771 | T1 | 2 | 0.08 | Clustered | AM(0.2) | 7 |
| 952 | 603777740003771 | CAS1_DELHI | 8 | 0.31 | Clustered | AA(0.4), AM(0.6) | 5, 11, 17, 18 |
| 954 | 703677740003771 | CAS | 1 | 0.04 | Unique | AA(0.2) | 19 |
| 966 | 775777777760771 | T | 1 | 0.04 | Unique | OR(0.1) | 21 |
| 1074 | 777777607760761 | LAM9 | 2 | 0.08 | Clustered | AM(0.2) | 6, 16 |
| 1077 | 777777377760731 | T2 | 2 | 0.08 | Clustered | AA(0.2), AM(0.1) | 3, 16 |
| 1081 | 777736777760731 | X1 | 1 | 0.04 | Unique | OR(0.1) | 6 |
| 1088 | 777767777763771 | MANU2 | 4 | 0.15 | Clustered | AA(0.2), AM(0.3) | 5, 7, 8 |
| 1094 | 777777777563771 | MANU2 | 1 | 0.04 | Unique | OR(0.1) | 4 |
| 1129 | 776777777760771 | T1 | 1 | 0.04 | Unique | OR(0.1) | 4 |
| 1134 | 777737777420731 | H3 | 1 | 0.04 | Unique | AM(0.1) | 12 |
| 1166 | 777377777760771 | T1 | 5 | 0.19 | Clustered | AA(0.2), AM(0.4) | 8, 12, 18, 19 |
| 1197 | 703777740203171 | CAS1_DELHI | 1 | 0.04 | Unique | SNNPR(0.4) | 21 |
| 1198 | 703737740003171 | CAS1_DELHI | 4 | 0.15 | Clustered | AM(0.3), OR(0.1) | 6, 8, 12 |
| 1199 | 703777740001171 | CAS | 2 | 0.08 | Clustered | AM(0.2) | 6, 11 |
| 1200 | 703777747777771 | F33 | 7 | 0.27 | Clustered | AM(0.8) | 6, 11, 12, 18 |
| 1227 | 777737557760771 | T1 | 1 | 0.04 | Unique | AM(0.1) | 12 |
| 1240 | 777760001760771 | T1 | 1 | 0.04 | Unique | AM(0.1) | 6 |
| 1264 | 703777740000000 | CAS1_DELHI | 7 | 0.27 | Clustered | AA(0.2), AM(0.3), AF(1.1), OR(0.3) | 3, 4, 6, 10, 16 |
| 1312 | 703777740003131 | CAS1_DELHI | 3 | 0.12 | Clustered | AM(0.1), OR(0.3) | 6, 12 |
| 1338 | 777777777777671 | U | 1 | 0.04 | Unique | AF(1.1) | 10 |
| 1360 | 577777777720631 | H3 | 1 | 0.04 | Unique | AM(0.1) | 12 |
| 1410 | 777777700000000 | U | 1 | 0.04 | Unique | SNNPR(0.4) | 6 |
| 1430 | 757777700003371 | H3 | 1 | 0.04 | Unique | AM(0.1) | 12 |
| 1457 | 777757777420771 | T1 | 3 | 0.12 | Clustered | AA(0.4), OR(0.1) | 5, 6 |
| 1530 | 777777607760711 | LAM9 | 1 | 0.04 | Unique | OR(0.1) | 6 |
| 1532 | 777776775760771 | X1 | 1 | 0.04 | Unique | AM(0.1) | 8 |
| 1547 | 777737777760771 | T3 | 2 | 0.08 | Clustered | AA(0.4) | 1 |
| 1551 | 701777740003771 | CAS1-Delhi | 4 | 0.15 | Clustered | AA(0.6), AM(0.1) | 5, 18 |
| 1552 | 777777774020631 | H1 | 2 | 0.08 | Clustered | AM(0.1), OR(0.1) | 4, 18 |
| 1580 | 777777764120771 | T1 | 1 | 0.04 | Unique | AM(0.1) | 7 |
| 1590 | 703777340003771 | CAS1-Delhi | 1 | 0.04 | Unique | OR(0.4) | 21 |
| 1624 | 776177607560771 | LAM3-LAM6 | 1 | 0.04 | Unique | AM(0.1) | 6 |
| 1634 | 777777777723771 | MANU2 | 3 | 0.12 | Clustered | AA(0.2), OR(0.1), SNNPR(0.4) | 5, 14, 21 |
| 1675 | 703367400001771 | CAS1-Kili | 3 | 0.12 | Clustered | OR(0.3), SNNPR(0.4) | 4, 6 |
| 1688 | 777777403760771 | T1 | 10 | 0.39 | Clustered | AA(0.2), AM(0.4), OR(0.7) | 3, 4, 6, 11, 18, 21 |
| 1689 | 777777607600031 | U | 1 | 0.04 | Unique | AA(0.2) | 3 |
| 1690 | 777777777762771 | MANU2 | 5 | 0.19 | Unique | AF(2.1), OR(0.4) | 14, 21 |
| 1729 | 700000004177771 | Ethiopian** | 32 | 1.23 | Clustered | AM(3.0), OR(0.4), SNNPR(0.4) | 6, 11, 12, 16, 18 |
| 1745 | 773737777760771 | T3 | 2 | 0.08 | Clustered | AM(0.2) | 11, 20 |
| 1787 | 703777740002171 | CAS1_DELHI | 2 | 0.08 | Clustered | AM(0.2) | 6, 16 |
| 1800 | 777777407760771 | LAM9 | 1 | 0.04 | Unique | AM(0.1) | 16 |
| 1821 | 777347777760771 | T1 | 15 | 0.58 | Clustered | AA(1.1), OR(1.3) | 5, 21 |
| 1877 | 737377777760771 | T1 | 8 | 0.31 | Clustered | AA(0.4), AM(0.3), OR(0.1), SNNPR(0.8) | 2, 6, 11, 18 |
| 1889 | 007777707760771 | T1 | 2 | 0.08 | Clustered | AA(0.2), AM(0.1) | 16, 17 |
| 1939 | 777777775720731 | H3 | 1 | 0.04 | Unique | AA(0.2) | 1 |
| 1952 | 777777774000771 | X1 | 1 | 0.04 | Unique | AA(0.2) | 19 |
| 1967 | 703777700003171 | CAS | 2 | 0.08 | Clustered | AF(2.1) | 10 |
| 2007 | 777737677760771 | T3 | 1 | 0.04 | Unique | AM(0.1) | 18 |
| 2102 | 703760000000771 | CAS | 1 | 0.04 | Unique | AA(0.2) | 19 |
| 2128 | 777707777760771 | T1 | 1 | 0.04 | Unique | AA(0.2) | 19 |
| 2306 | 777737770000771 | H1 | 1 | 0.04 | Unique | AM(0.1) | 16 |
| 2359 | 703677740003171 | CAS1_DELHI | 11 | 0.42 | Clustered | AM(1.1), OR(0.1) | 6, 18 |
| 2367 | 766377777760771 | T1 | 1 | 0.04 | Unique | OR(0.1) | 6 |
| 2392 | 703777700003171 | CAS | 1 | 0.04 | Unique | AA(0.2) | 19 |
| 2398 | 703777744037771 | F33 | 1 | 0.04 | Unique | AM(0.1) | 6 |
| 2409 | 777737757760771 | T3 | 4 | 0.15 | Clustered | AM(0.1), OR(0.3), SNNPR(0.4) | 6, 18 |
| 2551 | 777777607760701 | LAM9 | 1 | 0.04 | Unique | OR(0.1) | 6 |
| 2596 | 777737777760760 | T1 | 1 | 0.04 | Unique | OR(0.1) | 6 |
| 2683 | 703777600003171 | CAS | 1 | 0.04 | Unique | SNNPR(0.4) | 6 |
| 2692 | 777737707760771 | T1 | 1 | 0.04 | Unique | OR(0.1) | 6 |
| 2693 | 703763740003771 | CAS1_DELHI | 1 | 0.04 | Unique | AF(1.1) | 10 |
| 2731 | 777777777767771 | MANU2 | 5 | 0.19 | Clustered | OR(0.4), SNNPR(0.8) | 21 |
| 2781 | 777777777767771 | F33 | 1 | 0.04 | Unique | AF(1.1) | 10 |
| 2793 | 400000757760771 | T3 | 2 | 0.08 | Clustered | OR(0.3) | 6 |
| 2820 | 777777767760731 | T2 | 1 | 0.04 | Unique | AF(1.1) | 10 |
| 2973 | 703701740003171 | CAS | 4 | 0.15 | Clustered | AM(0.4) | 6 |
| 3026 | 703777700000071 | CAS | 1 | 0.04 | Unique | AM(0.1) | 6 |
| 3133 | 677777777420731 | T1 | 3 | 0.12 | Clustered | AM(0.1), OR(0.3) | 6 |
| 3134 | 777737377720771 | H3 | 49 | 1.89 | Clustered | AA(0.4), AM(0.3), OR(0.6), SNNPR(13.0) | 6, 18, 21 |
| 3135 | 777777470000000 | U (likely H) | 3 | 0.12 | Clustered | AM(0.1), SNNPR(0.8) | 6 |
| 3136 | 760000001760771 | T3-ETH | 1 | 0.04 | Unique | SNNPR(0.4) | 6 |
| 3137 | 776737777760771 | T1 | 15 | 0.58 | Clustered | AA(0.2), OR(1.6), SNNPR(1.2) | 6 |
| 3138 | 777776777700731 | T1 | 1 | 0.04 | Unique | OR(0.4) | 6 |
| 3139 | 577737777760771 | T1 | 2 | 0.08 | Clustered | OR(0.3) | 6 |
| 3140 | 776737777760731 | T1 | 1 | 0.04 | Unique | OR(0.1) | 6 |
| 3141 | 177000377760771 | T3 | 3 | 0.12 | Clustered | AM(0.1), OR(0.3) | 6 |
| 3203 | 777761000000000 | EAI1 | 1 | 0.04 | Unique | SNNPR(0.4) | 6 |
| 3309 | 777737777760571 | T1 | 2 | 0.08 | Clustered | OR(0.3) | 6 |
| 3313 | 000000004020631 | H3 | 1 | 0.04 | Unique | AM(0.1) | 6 |
| 3314 | 006737777760771 | T1 | 6 | 0.23 | Clustered | AA(0.2), AM(0.1), OR(0.4), SNNPR(0.4) | 6 |
| 3315 | 376777737760771 | T1 | 6 | 0.23 | Clustered | AA(0.4), OR(0.6) | 6 |
| 3316 | 377000377760771 | T3 | 1 | 0.04 | Unique | OR(0.1) | 6 |
| 3317 | 477737737760771 | T1 | 2 | 0.08 | Clustered | OR(0.3) | 6 |
| 3319 | 703417740003771 | CAS | 1 | 0.04 | Unique | SM(5.9) | 6 |
| 3320 | 743777777520731 | T1 | 2 | 0.08 | Clustered | OR(0.3) | 6 |
| 3321 | 757677777760771 | T1 | 5 | 0.19 | Clustered | OR(0.6), SM(5.9) | 6 |
| 3322 | 762737777760771 | T1 | 1 | 0.04 | Unique | OR(0.1) | 6 |
| 3323 | 776737377720771 | T1 | 2 | 0.08 | Clustered | SNNPR(0.8) | 6 |
| 3324 | 776737377760771 | T1 | 2 | 0.08 | Clustered | OR(0.3) | 6 |
| 3325 | 776737407760771 | LAM9 | 1 | 0.04 | Unique | OR(0.1) | 6 |
| 3326 | 377000377760771 | T3 | 2 | 0.08 | Clustered | OR(0.3) | 6 |
| 3327 | 777000377560771 | T3 | 3 | 0.12 | Clustered | AM(0.2), SM(5.9) | 6 |
| 3328 | 777000377760761 | T3 | 1 | 0.04 | Unique | OR(0.1) | 6 |
| 3329 | 777346777760601 | X2 | 1 | 0.04 | Unique | OR(0.1) | 6 |
| 3331 | 777737740160771 | LAM10 | 1 | 0.04 | Unique | SM(5.9) | 6 |
| 3332 | 777737740412771 | EAI4 | 2 | 0.08 | Clustered | OR(0.3) | 6 |
| 3334 | 737777777420731 | U | 2 | 0.08 | Clustered | SNNPR(0.8) | 6 |
| 3335 | 737777747413771 | EAI5 | 1 | 0.04 | Unique | SM(5.9) | 6 |
| 3336 | 700000007177770 | Ethiopian** | 2 | 0.08 | Clustered | SNNPR(0.8) | 6 |
| 3337 | 777737770360771 | T1 | 2 | 0.08 | Clustered | OR(0.3) | 6 |
| 3409 | 700020047177771 | Ethiopian** | 1 | 0.04 | Unique | AM(0.1) | 18 |
| 3411 | 777002377760771 | T3-ETH | 8 | 0.31 | Clustered | AM(0.9) | 18 |
| 3412 | 777003377760771 | T4 | 2 | 0.08 | Clustered | AM(0.2) | 18 |
| 3896 | 777737777760000 | T3 | 1 | 0.04 | Unique | AF(1.1) | 10 |
| 3897 | 777347777763671 | Manu2 | 3 | 0.12 | Clustered | AF(3.2) | 10 |
| 3898 | 677777077413771 | EAI2 | 1 | 0.04 | Unique | AF(1.1) | 10 |
| 3899 | 703777700001771 | CAS | 1 | 0.04 | Unique | AF(1.1) | 10 |
| 3900 | 777347777763771 | Manu2 | 5 | 0.19 | Clustered | AF(2.1), OR(0.3) | 10, 12 |
| 3902 | 777777457413771 | EAI6-BGD1 | 2 | 0.08 | Clustered | AF(2.1) | 10 |
| 3903 | 677777407760771 | LAM1 | 1 | 0.04 | Unique | AF(1.1) | 10 |

^a^ In the SITVIT2 database, the spoligo international type (SIT) numbers designate spoligotypes shared by two or more patient isolates. In contrast, “orphan” designates patterns reported for a single isolate. ^b^ Clade designations according to STVIT2 database: Beijing clade, East African-Indian (EAI) clade and 9 sub-lineages, Haarlem (H) clade and 3 sub-lineages, Latin American-Mediterranean (LAM) clade and 12 sub-lineages, the ancestral “Manu” family and 3 sub-lineages, the S clade, the IS6110-low-binding X clade and 3 sub-lineages, and an ill-defined T clade with 5 sub-lineages, U: Unknown patterns, ** Belongs to new Ethiopian L7. ^c^ AA: Addis Ababa; AM: Amhara; AF: Afar; BG: Benishangul Gumz; DD: Dire Dawa; GM: Gambela; HR: Harari; OR: Oromia; SNNPR: South Nation and Nationalities Peoples Region; SM: Ethiopian Somali; TG: Tigray ^d^High clustering rate reported.
